# Gene Expression Profiling of the Extracellular Matrix Signature in Macrophages of Different Activation Status: Relevance for Skin Wound Healing

**DOI:** 10.3390/ijms20205086

**Published:** 2019-10-14

**Authors:** Julia Etich, Manuel Koch, Raimund Wagener, Frank Zaucke, Mario Fabri, Bent Brachvogel

**Affiliations:** 1 Dr. Rolf M. Schwiete Research Unit for Osteoarthritis, Orthopaedic University Hospital Friedrichsheim gGmbH, 60528 Frankfurt/Main, Germany; 2 Center for Biochemistry, Medical Faculty, University of Cologne, 50931 Cologne, Germany; 3 Institute for Dental Research and Oral Musculoskeletal Biology, Medical Faculty, University of Cologne, 50931 Cologne, Germany; 4 Center for Molecular Medicine, University of Cologne, 50931 Cologne, Germany; 5 Department of Dermatology, University of Cologne, 50931 Cologne, Germany; 6 Department of Pediatrics and Adolescent Medicine, Experimental Neonatology, Medical Faculty, University of Cologne, 50931 Cologne, Germany; 7 Cologne Center for Musculoskeletal Biomechanics, University of Cologne, 50931 Cologne, Germany

**Keywords:** extracellular matrix, matrisome, pro- and anti-inflammatory macrophage, gene expression profiling, skin wound healing

## Abstract

The extracellular matrix (ECM) provides structural support for tissue architecture and is a major effector of cell behavior during skin repair and inflammation. Macrophages are involved in all stages of skin repair but only limited knowledge exists about macrophage-specific expression and regulation of ECM components. In this study, we used transcriptome profiling and bioinformatic analysis to define the unique expression of ECM-associated genes in cultured macrophages. Characterization of the matrisome revealed that most genes were constitutively expressed and that several genes were uniquely regulated upon interferon gamma (IFNγ) and dexamethasone stimulation. Among those core matrisome and matrisome-associated components transforming growth factor beta (TGFβ)-induced, matrix metalloproteinase 9 (MMP9), elastin microfibril interfacer (EMILIN)-1, netrin-1 and gliomedin were also present within the wound bed at time points that are characterized by profound macrophage infiltration. Hence, macrophages are a source of ECM components in vitro as well as during skin wound healing, and identification of these matrisome components is a first step to understand the role and therapeutic value of ECM components in macrophages and during wound healing.

## 1. Introduction

The extracellular matrix (ECM) provides structural support for cells and tissues, but also modulates cell differentiation, activation and migration. For instance, components of the trabecular bone extracellular matrix support the formation of the hematopoietic niche. We and others could show that loss of ECM components in the trabecular bone impairs hematopoietic stem-cell differentiation and immune-cell activation [1,2,3]. Inflammation is an important aspect of any tissue injury and recent findings show that activated macrophages are key regulators during skin wound healing [4,5]. Macrophages infiltrate the damaged skin and in response to the extracellular matrix, including native ECM molecules and their degradation products, became activated and polarized [6,7,8] to phagocytose and kill pathogens during early inflammation. Later during remodeling, macrophages remove dead cells and suppress further activation of immune cells in the wound to resolve the inflammation. These anti-inflammatory macrophages also remodel the ECM by secreting proteases and this remodeling is needed to orchestrate the wound-healing process [9]. Dysregulated activation of macrophages during tissue repair can result in chronic inflammation characterized by excessive deposition of ECM severely impairing tissue architecture and function in chronic wounds or fibrosis [10]. 

Within the wound, macrophages produce soluble mediators, such as transforming growth factor beta (TGFβ), platelet-derived growth factor (PDGF) and insulin-like growth factor (IGF1), that stimulate local and recruited tissue fibroblasts to differentiate into myofibroblasts and to promote transition from inflammation to regeneration in skin tissue repair [11,12]. Recently, it was shown that macrophages in response to IL-4 polarization regulate the formation of vascular structures in skin wounds. They also control collagen fibril formation by inducing the activity of the collagen modifying enzyme lysyl hydroxylase 2 in adjacent fibroblasts which in turn facilitates cross-link formation [13]. Such interactions promote wound closure as well as the synthesis of ECM components and we have shown that myofibroblasts display a unique and specific expression profile at the peak of in situ granulation tissue formation [14]. However, macrophages can also regulate wound healing independently of their interactions with (myo-)fibroblasts. While macrophage-derived molecules are known to directly promote ECM disruption and destabilization, it is increasingly appreciated that they also contribute to ECM formation, maintenance, and function. Earlier it was reported that monocytes and macrophages can express several collagens, and among those collagens VI, VIII and XXIII were suggested to promote tissue integrity or cell-matrix and cell–cell interactions [15,16,17]. Moreover, macrophages synthesize proteoglycans that participate in the formation, stabilization and function of a hyaluronan-rich ECM [18] suggesting a central role for macrophages in matrix reorganization during tissue repair. 

Hence, macrophages may provide important ECM-associated components to orchestrate the tissue repair process, but limited knowledge exists about the production and regulation of ECM molecules in activated macrophages. We used a previously published mRNA transcriptome of human macrophages [19] to define the expression of genes encoding ECM proteins and ECM-associated components. We also analyzed the pro- and anti-inflammatory response of the core matrisome and matrisome-associated molecules after Interferon gamma (IFNγ) and dexamethasone stimulation. Moreover, we could show that matrisome candidates that were identified from in vitro analysis are found in the in vivo wound at the peak of macrophage infiltration. These matrisome components could be important not only for the activation of macrophages, but also for the structural support of the wound microenvironment. In addition to the ability of macrophages to promote wound healing by interacting and influencing myofibroblasts, wound macrophages may directly modulate the transition from inflammation to proliferation and remodeling. 

## 2. Results

### 2.1. Core Matrisome and Matrisome-Associated Genes Are Expressed in Human Macrophages

**Figure 1:**
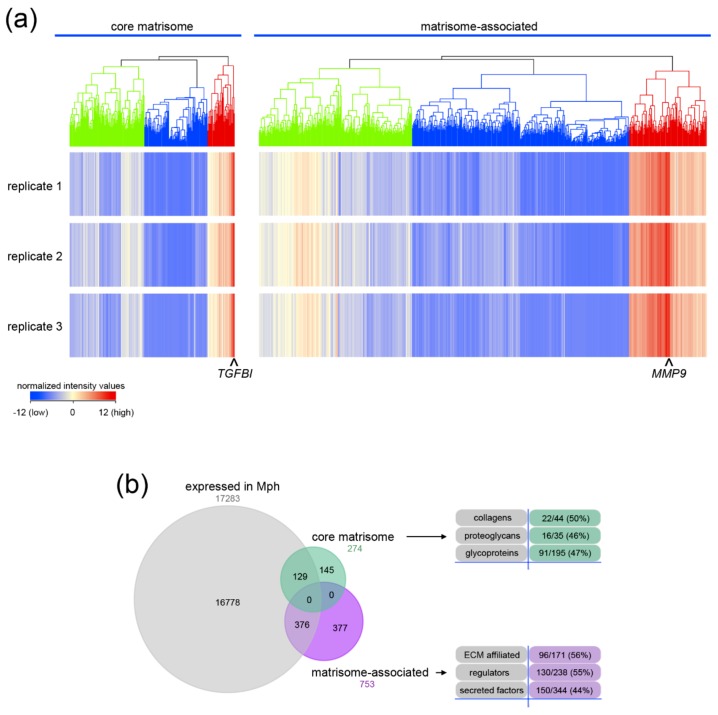
Transcriptome analysis of the matrisome of primary human macrophages. (**a**) Non-averaged hierarchical clustered intensity plot (distant metrics–euclidean, linkage rule–ward’s) of core matrisome and matrisome-associated genes in macrophages is shown. Clusters of intermediate (green), low (blue) and high (red) expression are highlighted. The normalized intensity values of the individual replicates are shown. The highest expressed gene within the core matrisome *TGFBI* (transforming growth factor beta induced) and the matrisome-associated *MMP9* (matrix metalloproteinase 9) are indicated. (**b**) The proportion of entities within the core matrisome or matrisome-associated cluster are shown in a Venn diagram. The numbers and percentages of regulated genes found in subcategories are listed.

**Figure 2:**
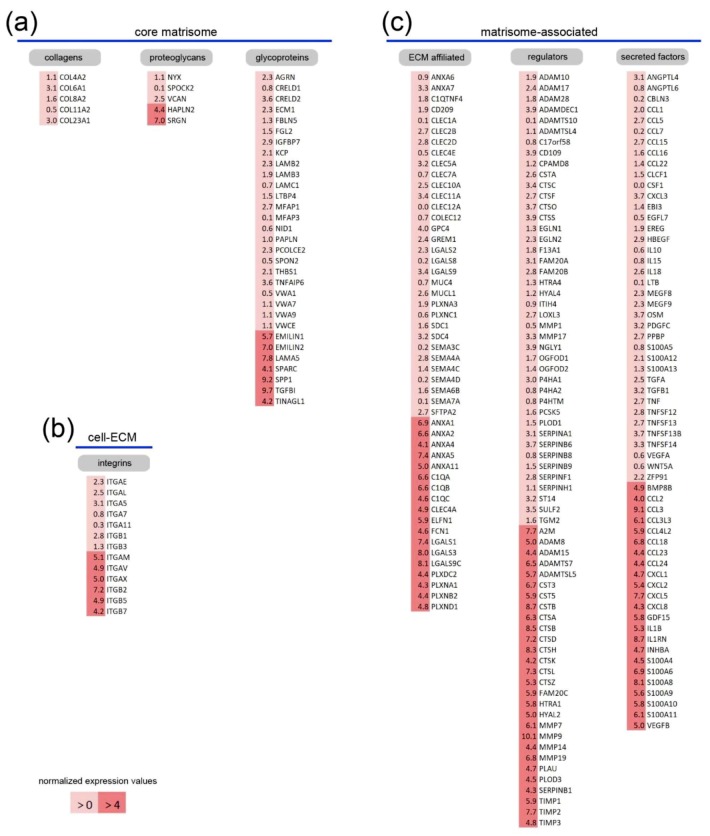
Identification of highly expressed core matrisome and matrisome-associated genes in primary human macrophages. The most highly expressed genes of the core matrisome (**a**), cell–matrix interaction-mediating integrin (**b**) and matrisome-associated genes (**c**) are listed according to intermediate (light red) and high (red) expression. Normalized expression values are given and the complete set of genes with respective expression values is given in the Table S1.

The transcriptome of isolated monocyte-derived macrophages (MDMs) was used to analyze the expression of the core matrisome and matrisome-associated genes (http://matrisomeproject.mit.edu) [20,21]. Hierarchical cluster analysis identified a cluster of intermediately, lowly and highly expressed genes for each category (Figure 1a). Transforming growth factor β induced protein (*TGFBI*) and matrix metalloproteinase 9 (*MMP9*) were the most strongly up-regulated genes of the core matrisome and matrisome-associated genes, respectively. 129 of 274 genes of the core matrisome and 376 of 753 genes of the matrisome-associated cluster were expressed in macrophages (Figure 1b). The transcripts of 22 collagen α-chains (50%) as well as 16 proteoglycan core proteins (46%) and 91 glycoproteins (47%) were expressed in MDMs. A high proportion of matrisome-associated genes were also expressed in MDMs. The transcripts of 96 ECM-affiliated genes (56%), 130 ECM regulators (55%) and 150 secreted factors (44%) were detected in MDMs. Genes with a moderate to high expression were analyzed in detail (Figure 2).

Among genes encoding members of the collagen family only the network-forming *COL4A2*, *COL6A1* and *COL8A2*, the fibril-forming *COL11A2* and the membrane-bound *COL23A1* were moderately expressed (Figure 2a). Other chains and collagen genes were expressed at lower levels (Table S1). Five proteoglycan-related genes were expressed at higher levels and serglycin (*SRGN*) and hyaluronan and proteoglycan link protein 2 (*HAPLN2*) were most strongly expressed in MDMs. Notably, two other hyaluronan and proteoglycan link proteins (HAPLN3 and HAPLN4) were expressed in MDMs at lower levels (Table S1). Link proteins are known to stabilize the interaction of hyluronan and lecticans and, interestingly, the lectican versican (*VCAN*) was moderately expressed in macrophages. Glycoproteins represent the largest cluster within the core matrisome and 24 genes were expressed at moderate levels while for seven genes high expression was detected. Remarkably, four laminin chains (*LAMA5*, *LAMB2*, *LAMB3*, *LAMC1*), three of which can assemble into the mature laminin-521, were expressed at moderate or high levels. *EMILIN1*, *EMILIN2*, *LAMA5*, *SPARC*, *SPP1*, *TGFBI* and *TINAGL1* formed the group of glycoproteins with the highest expression in macrophages and all of them have been reported to interact with integrins [22,23,24,25,26,27]. Interestingly, many of the known integrin genes were also expressed in MDMs (Table S1) and the highest expression was for *ITGAM*, *ITGAV*, *ITGAX*, *ITGB2*, *ITGB5* and *ITGB7* (Figure 2b).

Many matrisome-associated genes were expressed in macrophages and 32 ECM-affiliated genes were moderately while 18 strongly expressed in MDMs (Figure 2c). Those included five genes for calcium-regulated phospholipid-binding annexins (*ANXA1*, *ANXA2*, *ANXA4*, *ANXA5*, *ANXA11*), three genes of the complement cascade (*C1QA*, *C1QB* and *C1QC*) and the extracellular leucine rich repeat and fibronectin type III domain containing 1 (*ELFN1*). Several genes for lectins were also among those most highly expressed (*CLEC4A*, *FCN1*, *LGALS1*, *LGALS3*, *LGALS9C*). In addition, some genes of the plexin family (*PLXDC2*, *PLXNA1*, *PLXNB2*, *PLXND1*), whose members act as receptors for semaphorin family signaling proteins [28,29], were highly expressed and, interestingly, many of the semaphorin genes were expressed in MDMs (*SEMA3A* to *SEMA7A*). 

Proteolytic enzymes secreted by immune cells cleave ECM proteins leading to altered physical and biochemical properties of the tissue [30]. Such extracellular proteases and their inhibitors are found in the group of regulators of the matrisome-associated cluster and 42 were intermediately and 28 highly expressed. Proteases form one of the largest and most diverse families of enzymes known and control of their activity is essential to limit cleavage to intended substrates only. The secreted serine protease urokinase-plasminogen activator (*PLAU*), matrix metalloproteinases (*MMP7*, *MMP9*, *MMP14*, *MMP19*), two ADAMs (*ADAM8*, *ADAM15*) as well as *ADAMTS7* and *ADAMTSL5* were among the highest expressed proteases. Moreover, inhibitors of matrix metalloproteinases are highly expressed in MDMs, such as the proteinase-entrapping alpha-2-macroglobulins (*A2M*) or tissue inhibitors of metalloproteinases (*TIMP1*, *TIMP2*, *TIMP3*) known to strictly control metalloprotease pro- and anti-inflammatory activity. Several serine, aspartyl and cysteine cathepsins (*CTSA*, *CTSD*, *CTSB*, *CTSH*, *CTSK*, *CTSL*, *CTSZ*) as well as corresponding cysteine protease inhibitors, the cystatins (*CST3*, *CST5*, *CSTB*), were strongly expressed. Furthermore, serine proteases (*FAM20C*, *HTRA1*) and the serine protease inhibitor *SERPINB1* were highly expressed in MDMs. The glycosylphosphatidylinositol-anchored hyaluronidase (*HYAL2*) which degrades the hyaluronan (HA)-containing pro-inflammatory matrix in concert with the classical HA receptor CD44 [31] was one of the highly expressed genes. Similarly, *PLOD3*, the gene encoding the collagen-modifying lysyl hydroxylase 3 (LH3), was strongly expressed.

Secreted factors that are sequestered in the ECM to deposit cell activation and differentiation signals in the local environment belong to the matrisome-associated components. Within the cluster of secreted factors, MDMs expressed genes for various chemokines (*CCL2*, *CCL3*, *CCL3L3*, *CCL4L2*, *CCL18*, *CCL23*, *CCL24*, *CXCL1*, *CXCL2*, *CXCL5*, *CXCL8*) and growth factors of the TGFβ and the vascular-endothelial growth factor (VEGF) families (*BMP8B*, *GDF15*, *INHBA*, *VEGFB*) that are important for the recruitment and activation of myeloid cells. In addition, interleukin 1β (*IL1B*), interleukin 1 receptor antagonist (*IL1RN*) and several genes encoding S100 proteins were highly expressed in MDMs (*S100A4*, *S100A6*, *S100A8*, *S100A9*, *S100A10*, *S100A11*). In summary, human MDMs express a network of genes associated with ECM production, cell-matrix interaction, ECM degradation and cell communication. 

### 2.2. A Unique Panel of Core Matrisome and Matrisome-Associated Genes Is Regulated in Interferon Gamma (IFNγ)- and Dexamethasone-Primed Macrophages

**Figure 3:**
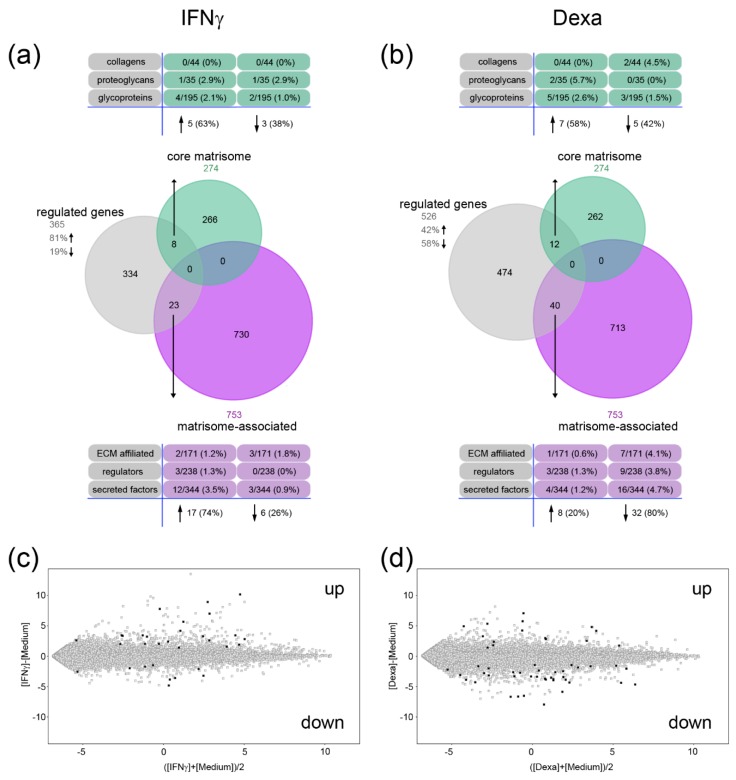
Regulation of core matrisome and matrisome-associated genes after interferon gamma (IFNγ) and dexamethasone (Dexa) stimulation. (**a**,**b**) The proportion of entities among the regulated genes in IFNγ- (**a**) or dexamethasone-primed macrophages (**b**) that are found in the core matrisome or matrisome-associated data set are shown in a Venn diagram. The numbers and percentages of genes in the subcategories are listed. (**c**,**d**) Expression intensity plots for IFNγ- (**c**) or dexamethasone-primed macrophages (**d**) are shown. Regulated genes within the core matrisome and the matrisome-associated cluster are highlighted in black. (fold change ≥ 2, *p* < 0.05, false discovery rate (FDR) correction by Benjamini-Hochberg, medium versus IFNγ or medium versus dexamethasone).

Macrophages respond to the microenvironment of the wound and can change their pro-inflammatory phenotype to an anti-inflammatory phenotype to facilitate the transition from the inflammation phase to the proliferation and remodeling phase of wound healing. Changes in activation could be accompanied by changes in ECM expression and we analyzed the transcriptional changes of the matrisome in reponse to the pro-inflammatory IFNγ and the anti-inflammatory dexamethasone. Entities that show a signal intensity above background noise and a significant change in relative expression levels (fold change (FC) ≥ 2, *p*-value ≤ 0.05, false discovery rate (FDR) by Benjamini-Hochberg) were considered to be differentially expressed between the conditions. In IFNγ-stimulated macrophages, a total of 365 transcripts were significantly regulated compared to control MDMs and 81% of these were up- and 19% were down-regulated (Figure 3a). Among these, five genes of the core matrisome were up- (63%) and three down-regulated (38%). Interestingly, the expression of matrisome-associated genes was mainly increased after IFNγ stimulation (17 genes, 74%) (Figure 3a). In general, the matrisome was more responsive to the anti-inflammatory stimulus of dexamethasone. A total of 526 transcripts were differentially expressed upon such treatment and 42% of these were increased and 58% decreased in their expression. Several core matrisome genes were up- (7 genes, 58%) and down-regulated (5 genes, 42%), while the majority of matrisome-associated genes were significantly down-regulated (32 genes, 80%) (Figure 3b). Hence, IFNγ induced a pronounced upregulation (Figure 3c) and dexamethasone a downregulation of the matrisome gene expression (Figure 3d). 

**Figure 4:**
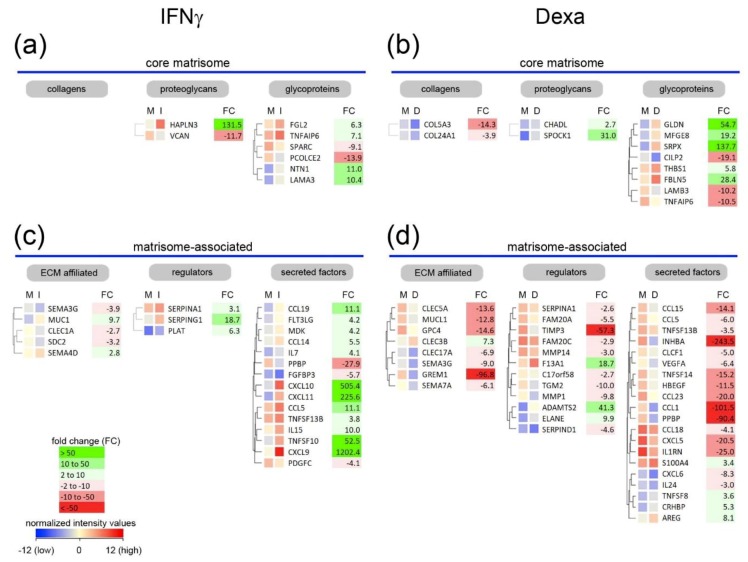
Cluster analysis of regulated matrisome genes in macrophages stimulated with IFNγ or dexamethasone. (**a**–**d**) Clustered genes of the core matrisome (**a**,**b**) and matrisome-associated cluster (**c**,**d**) regulated in monocyte-derived macrophages (MDMs) after IFNγ (**a,c**) or dexamethasone stimulation (**b**,**d**, Dexa) are displayed. Non-averaged hierarchical clustered intensity plot (distant metrics–euclidean, linkage rule–ward’s) of differentially expressed genes in cells cultured in normal medium (M) and medium containing IFNγ (I) or dexamethasone (D) is shown and the fold change (FC) is given.

Next, differentially expressed genes were clustered according to their similarity in expression within each category of the matrisome (Figure 4, Table S1). Within the group of proteoglycan-related genes, *HAPLN3* was strongly induced upon pro-inflammatory stimulation by IFNγ (Figure 4a) and was even more highly expressed than *HAPLN2*. The latter was one of the most highly expressed proteoglycan-related genes in unstimulated macrophages (Figure 2a). HAPLNs are known to mediate and stabilize the interaction of lecticans with hyaluronan. In the larger cluster of glycoproteins, netrin-1 (*NTN1*) and the laminin α3 chain (*LAMA3*) genes were more than 10-fold up-regulated in pro-inflammatory macrophages. 

Treatment of MDMs with dexamethasone reduced the expression of most of the matrisome-associated genes and only few were increased (Figure 4d). The metalloproteinase *ADAMTS2* (41-fold), the transglutaminase coagulation factor XIII A chain (*F13A1*, 19-fold) and the neutrophil elastase (*ELANE*, 10-fold) were up-regulated, while the tissue inhibitor of metalloproteinases 3 (*TIMP3*) was 57-fold down-regulated. Within the group of secreted factors, most genes were moderately down-regulated but the expression of *INHBA*, *CCL1* and *PPBP* was at least 90-fold decreased and only the expression of *S100A4*, *TNFSF8*, *CRHBP* and *AREG* was slightly increased.

**Figure 5:**
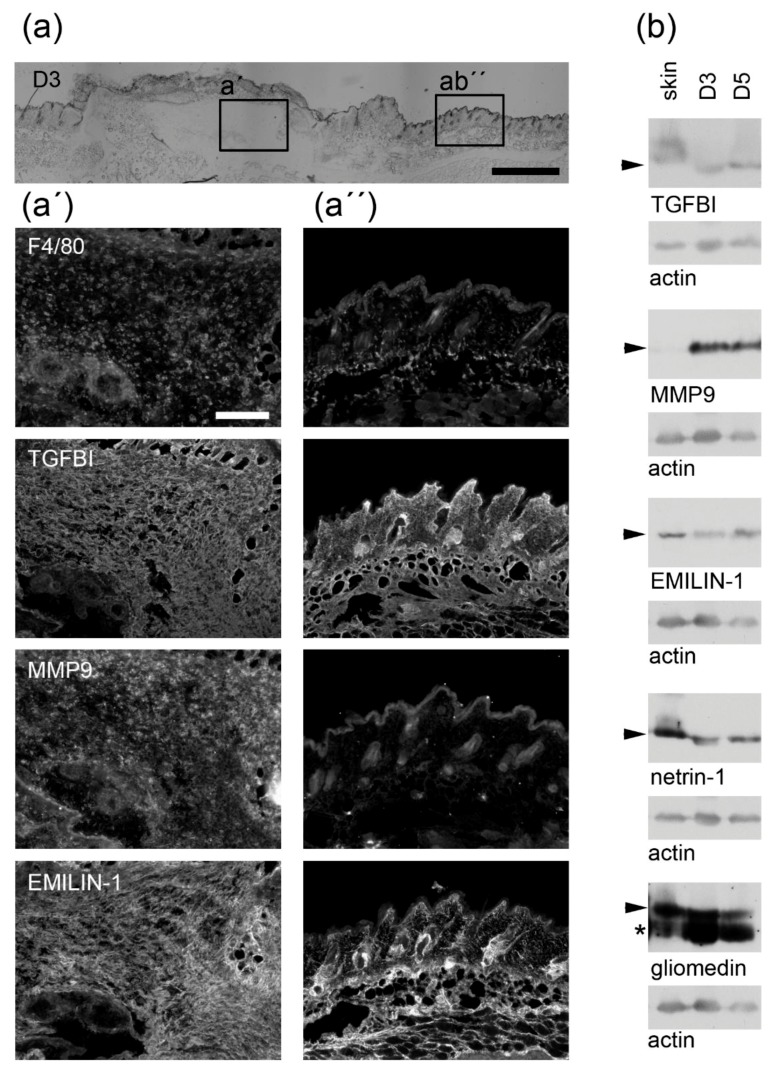
Validation of matrisome proteins in the in vivo wound bed in a murine wound healing model. (**a**) In wounds that were harvested three days post wounding the macrophage-specific F4/80 as well as transforming growth factor beta induced (TGFBI), MMP9 and EMILIN-1 were detected by immunofluorescence analysis within the wound (**a’**) or the intact skin (**a´´**). (**b**) TGFBI, MMP9, EMILIN-1, netrin-1 and gliomedin abundance (arrowhead) was analyzed by immunoblotting in lysates of intact skin (skin) or wounds three (D3) and five days post wounding (D5). Actin was used as loading control. As EMILIN-1 and gliomedin were tested on a single blot, the same actin control is included in both panels. Molecular weights of Thermo Scientific™ PageRuler™ Plus Prestained 10–250 kDa Protein Ladder bands are given. *, unspecific band; scale bars (**a**), 1000 µm; (**a´**, **a´´**), 200 µm.

To demonstrate that ECM components could be important for macrophage-modulated wound healing, immunofluorescence and immunoblot analysis of mouse skin wounds at the peak of macrophage infiltration were performed. At early time points of the healing process, the wound is mainly repopulated by hematopoietic cells [32]. At day three and five post wounding the majority of the cells are macrophages whereas fibroblasts, vascular cells, neutrophils or platelets are hardly found within the wound bed [14,32,33]. We confirmed by immunofluorescence studies that at day three post wounding F4/80^+^ macrophages are found within the wound bed (Figure 5a). Interestingly, at this time point TGFBI and MMP9, both highly expressed in isolated macrophages, were also detected in the wound (Figure 5a,b). While TGFBI was also found in intact skin, MMP9 was absent. In addition, the constitutively expressed EMILIN-1, the IFNγ-induced netrin-1 and dexamethasone-induced gliomedin were detected not only in the intact skin, but also within the wound (Figure 5a,b).

In summary, a panel of core matrisome and matrisome-associated genes were expressed in macrophages. Most genes were constitutively expressed, but several core matrisome genes were uniquely regulated upon IFNγ and dexamethasone stimulation of MDMs. IFNγ stimulation predominantly induced the expression, while dexamethasone stimulation inhibited the expression of matrisome-associated genes. In addition, encoded proteins of several candidate genes were found in the in vivo wound bed at time points characterized by macrophage invasion. Hence, the data presented in this study provide insight into a tightly regulated macrophage-specific extracellular matrix signature that can be used for targeting analyses of ECM-specific genes/gene networks in wound beds in vivo.

## 3. Discussion

Macrophages are essential regulators of inflammation and tissue remodeling. They adaptively change their function depending on the extracellular microenvironments. One major goal of our study was to characterize the expression of extracellular matrix genes by human macrophages that may be relevant for skin-tissue repair. Due to inavailability of normal healing wounds for research purposes, human wound macrophages cannot be isolated in sufficient amounts and, therefore, we used microarray data of in vitro differentiated human monocyte-derived macrophages, which were treated with IFNγ, a classical pro-inflammatory stimulus, dexamethasone, the prototypical anti-inflammatory compound, or left untreated. We could identify candidate genes encoding the core matrisome and matrisome-associated proteins that are expressed by macrophages. Moreover, we defined distinct patterns of gene expression induced by IFNγ and dexamethasone. Several of the identified candidates could be detected in the in vivo wound at a time point when macrophages are the main cellular component of the wound. Thereby, we identified potential key regulators of macrophage behavior at distinct activation statuses that are relevant for the skin wounding response. 

### 3.1. Macrophages Could Provide Structural Integrity by Expressing Core Matrisome Genes

For the core matrisome, we could confirm that genes encoding collagens VI [16], VIII [17] and XXIII [15] are significantly expressed in macrophages. While collagens in general contribute to ECM structure, macrophage-derived collagen VI was shown to also be captured at the cell surface and to bridge cell-cell interactions [16]. This indicates that endogenous collagens can modulate the behavior of macrophages. We have identified two more collagen genes (*COL11A2*, *COL4A2*) that are moderately expressed in macrophages. Collagen XI is a minor fibril constituent in cartilaginous tissues that mainly contain collagen II [34], but has also been reported to be expressed in non-cartilaginous tissues [35]. Together with collagen V, collagen XI comprises a subclass of regulatory fibrillar collagens that co-assemble with collagens I, II and III to control lateral growth of collagen fibrils [36]. Macrophages display strong affinity for collagen fibrils and promote collagen fibrillogenesis at the terminal end bud in the mammary gland [37]. Accelerated biosynthesis of collagen and its fibril formation is required for proper wound healing, whereas excessive accumulation of collagen is the hallmark of fibrotic diseases. Considering the ability of macrophages to capture collagens at their cell surface, collagen XI may be utilized by macrophages to interact with fibrillar collagens. Thereby, macrophages may support organization of collagens that are secreted by myofibroblasts, which differentiate from wound-resident fibroblasts or from other progenitor cells in the wound [38]. 

Collagen IV is a constituent of basement membranes and is indispensable for the structural integrity and functions of these specialized, self-assembled extracellular matrices [39]. It was shown that macrophages directly interact with the developing vasculature during angiogenesis [40] and might remodel native basement membrane barriers in a MMP14-dependent fashion [41]. Our results indicate that macrophages are also a source of collagen IV and might contribute to the integrity of vascular and skin basement membranes. Laminins form the other main structural element of basement membranes and are found in the glycoprotein category within the core matrisome. Four laminin chains were among the highest expressed glycoprotein genes in macrophages, three of which form the laminin-521. The laminin α5 chain, found not only in laminin-521 but also in laminin-511, is expressed in the vascular basement membrane as well as in the basement membrane underlying the interfollicular epidermis in the skin and promotes angiogenesis and re-epithelialization [42,43]. Collagen IV and laminins play essential roles in the basement membrane formation and stability via self-interactions and interactions with other components [44] as well as in basement membrane repair [45]. Thus, macrophages could produce collagen IV, laminins and integrins to provide their own substrate and/or bridging proteins for their adhesion to basement memranes. Interestingly, loss of every of the most strongly expressed glycoprotein genes (*EMILIN1*, *SPP1*/osteopontin, *SPARC*/osteonectin,) in mice leads to accelerated wound closure and/or altered matrix organization [46]. EMILINs as components of elastic fibers are found in regions where elastin and fibrillin microfibrils are in close contact and in vivo these glycoproteins are exclusively targeted to fibrillin microfibrils within the wound [47]. Therefore, macrophages may produce EMILINs to regulate elastic fibre formation. Interestingly, collagen IV, VI, XI and elastin were shown to interact with the heparansulfate chains of perlecan providing ECM and pericellular matrix stabilization as well as organization [48,49,50,51]. 

Two structurally related glycoprotein genes (*LAMA3*, *NTN1*) were hardly expressed in unstimulated macrophages, but were induced upon IFNγ stimulation. As structural scaffold proteins, laminins are essential to tissue architecture and stability and the α3 chain is known to be incorporated into laminin-311, -321 and -322 to bind growth factors from the VEGF/PDGF, fibroblast growth factor (FGF), bone morphogenetic protein (BMP), and neurotrophin families [52]. Thereby, laminins may sequester these growth factors in the ECM to stimulate various cells within the wound and promote tissue regeneration and remodeling upon release [53]. Netrin-1 was initially described to control guidance of commissural axons in the central nervous system [54]. Since then, netrin-1 was reported to play key roles also in immune cell migration, angiogenesis, and cell survival [55] via interaction with its main receptors, the uncoordinated locomotion 5 (UNC5) homologs [56]. Recently, netrin-1 was shown to promote epithelial migration and resolution of inflammation during diabetic corneal wound healing [57]. The expression of netrin-1 in IFNγ stimulated macrophages and its detection in the macrophage-enriched wound tissue indicates that macrophages may provide guidance signals to organize the wound and induce the transition from inflammation to proliferation and subsequent remodeling. Macrophages, by expressing numerous genes encoding non-collagenous matrix proteins, may therefore be actively involved in the organization of the extracellular matrix environment in wounds. 

### 3.2. Distinct Core Matrisome Genes That Are Induced in Dexamethasone-Stimulated Macrophages Contribute to Re-Epithelization and Neovascularization

When macrophages were stimulated with dexamethasone, the two strongly induced core matrisome genes *GLDN* and *SRPX* could be identified the first of which could also be detected in the in vivo wound tissue. Gliomedin (*GLDN*) plays an important role in the formation and maintenance of the nodes of Ranvier on myelinated axons in the central nervous system. This glycoprotein is bound and clustered by perlecan that itself is recruited by dystroglycan to nodes of Ranvier [58]. Perlecan, a heparan sulfate proteoglycan, was originally identified in basement membranes [59]. Thus, macrophage-derived gliomedin might participate in basement-membrane formation/stabilization during wound healing process and influence keratinocytes to regulate epithelial wound closure. 

Interestingly, testican-1 (*SPOCK1*) was also induced in dexamethasone-stimulated macrophages. Similar to other proteoglycans [60,61,62], testican-1 and its glycosaminoglycan side chains might be capable of controlling diverse cellular behaviours including proliferation, differentiation, migration and matrix synthesis in repair processes. *SPOCK1* is a target of TGF-β and induces epithelial-to-mesenchymal transition (EMT) in lung cancer [63]. During wound healing, aspects of EMT are reflected in the epithelial wound closure which is required to restore the physical barrier of the skin [64]. The ability of immune cells to interact with and regulate keratinocytes has been described decades ago [65] and anti-inflammatory macrophages that are found at later stages of the wound-healing process can apparently produce ECM molecules to support re-epithelization. Testican-1 was also shown to promote corneal wound healing by modulating MMP-2 activation in vivo [66] and macrophage-derived testican-1 may regulate metalloproteinase activity to promote the degradation of provisional wound matrix to support vascularization. *FBLN5* was the only gene of the fibulin family that was significantly expressed in macrophages and further up-regulated upon dexamethasone stimulation. Fibulin-5 is essential for elastic fiber formation and for stabilization and organization of elastic fibers in skin. Consequently, *FBLN5*-deficient mice develop a phenotype which resembles the human cutis laxa syndrome indicating that fibulin-5 acts as a scaffold protein that organizes and links elastic fibres to the cells [67,68]. In addition, fibulin-5 promotes adhesion of endothelial cells through interaction with integrins [69], and inhibits angiogenesis and endothelial cell activities by antagonizing VEGF signaling independent of its integrin-binding properties [70]. Thus, fibulin-5 may be synthesized by anti-inflammatory macrophages to control neovascularization within the wound healing process. 

### 3.3. Macrophages Express Genes That Are Involved in the Release of Bioactive Fragments from the Extracellular Matrix (ECM)

A number of cell types contribute to the proteolytic environment within the wound but invading macrophages are considered the major source of proteins with enzymatic activity [71]. Many of the genes encoding metalloproteinases as well as the corresponding inhibitors were constitutively expressed in unstimulated macrophages. Remarkably, *ADAMTS2* gene expression was strongly induced and *TIMP3* expression strongly repressed in dexamethasone-stimulated macrophages. TIMP3 is a wide-spectrum inhibitor with activity towards several MMPs, a disintegrin and metalloproteinases (ADAMs) and ADAMs with thrombospondin motifs (ADAMTSs), including ADAMTS2 [72]. ADAMTS2 is a pro-collagen N-propeptidase that processes the aminopropeptide of fibrillar collagens I and III in skin. That is important for the deposition of normal collagen fibrils in the ECM as well as for their turnover [73]. In addition, proteins of the core matrisome and the matrisome-associated cluster have been identified as ADAMTS2 substrates, such as agrin (AGRN), MMP1, tissue inhibitor of metalloproteinases 1 (TIMP1), versican (VCAN), lectin, galactoside-binding, soluble (LGALS1, galectin-1) and inhibin subunit beta A (INHBA) [74]. All of these were moderately to highly transcribed by macrophages and/or regulated upon pro- and anti-inflammatory stimulation (Figure 2 and Figure 4). Degradation of the ECM can liberate bioactive fragments from proteins that otherwise provide structural support to the tissue. Such bioactive fragments are released from full-length proteins of the core matrisome, ECM-affiliated proteins and ECM regulators by limited proteolysis catalyzed by a variety of enzymes such as cathepsins, metalloproteases or furin type proprotein convertases [75]. These so called “matrikines” signal directly from the extracellular environment and “matricryptins” require proteolytic processing to reveal the ligand or to release the ligand from its ECM protein; both have been linked to cutaneous cancers and skin repair [76]. The anti-angiogenic matrikine endostatin, a C-terminal proteolytic fragment of collagen XVIII can be generated by *MMP7*, *MMP9* [77] and *CTSL* [78] all of which were constitutively high expressed in MDMs, as well as by the neutrophil elastase (*ELANE*) [79], that was induced by dexamethasone treatment. Several ECM proteins harboring matrikines/matricryptins are also strongly expressed in macrophages and/or regulated upon pro- and anti-inflammatory stimulation: Endotrophin, originally identified as an adipokine, is released by C-terminal cleavage of the collagen VI α3 chain. This matrikine not only augments fibrosis, angiogenesis, and inflammation through recruitment of macrophages and endothelial cells [80], but also enhances EMT in human breast cancer cells [81]. Only very recently, the metalloproteinase BMP-1 and proprotein convertases were identified as key players in the release of endotrophin and endotrophin-containing fragments [82]. Collagen IV is cleaved into several fragments with similar anti-angiogenic activities [83,84,85,86,87,88] while secreted protein acidic and rich in cysteines (SPARC)/osteonectin cleavage generates both anti-angiogenic and pro-angiogenic fragments [89]. The major heparin binding sequence in the LG4 domain of the laminin α3 chain promotes early-stage wound healing by reducing inflammation, accelerating re-epithelialization and decreasing granulation tissue formation [90]. Thus, macrophages express proteases to generate bioactive ECM fragments during the different stages of skin repair and also are a source of ECM molecules that harbor these bioactive fragments. 

## 4. Materials and Methods 

### 4.1. Bioinformatic Analysis

Macrophage gene expression files used in this study are available under accession no. GSE79077 in the Gene Expression Omnibus database (http://www.ncbi.nlm.nih.gov/geo/). Human C14^+^ blood monocytes were previously differentiated to macrophages by M-CSF for 4 to 7 days, cultured with medium alone, IFNγ or dexamethasone for 20 h and analyzed in biological triplicates using the Sureprint G3 human GE 8 × 60 K whole genome mRNA microarray [19]. The data were imported in GeneSpring 14.9 (Agilent Technologies, Hamburg, Germany) to create an Agilent Single Colour Experiment. For normalization the threshold raw signals were set to 1.0, percentile shift was used as normalization algorithm (75^th^ percentile) and no baseline transformation was performed. For Analysis a gene-level experiment was created where entities where at least 100.0% of samples in any 1 out of 1 conditions have flags in. Matrisome gene clusters, annotated in the matrisome database 2.0 (http://matrisomeproject.mit.edu) were imported and selected for hierarchical cluster analysis (distant metrics–euclidean, linkage rule–Ward’s) to determine relationships among the expression levels. To determine constitutively expressed and regulated genes in the set of unstimulated as well as IFNγ or dexamethasone-stimulated human MDMs the data was filtered on expression (20–100) th percentile in the raw data and on error -CV < 50 percent. A fold change cut off (FC ≥ 2) and moderated t-test cut off (*p*-value ≤ 0.05) was used to define differentially expressed mRNAs. False discovery rate was adjusted using Benjamini-Hochberg procedure. Expression intensity plots (MvA plots) were generated by highlighting regulated matrisome genes within the entire entities. The entity lists were exported to generate graphical representations as Venn diagrams using the FunRich 3.1 tool [91]. 

### 4.2. Wound-Healing Experiments

Animal experiments were performed with C57BL/6N mice in accordance with the animal ethics guidelines of the German law. Institutional review board: “Landesamt für Natur, Umwelt- und Verbraucherschutz NRW” (ethics approval no.: 2014.A012, approval date: 11 July 2014; ethics approval no.: 4.16.003, approval date: 18 February 2016). Full thickness wounds were inflicted on the back of C57BL/6N mice as described earlier [92]. Three and five days post injury wounds were embedded in tissue tek (Sakura Finetek Europe, Staufen im Breisgau, Germany), sectioned (Leica Cryotome CM3050, Wetzlar, Germany) and analyzed by immunofluorescence microscopy (Nikon Europe Eclipse TE2000-U Microscope, Tokyo, Japan) or the wound area was cropped using a fresh 6mm biopsy punch, underlying fat tissue was removed and the tissue was stored at −80 °C for up to two years.

### 4.3. Antibody Generation

A purified recombinant EMILIN-1 fragment (G173–G815) [47] or full length TGFBI was used for rabbit immunization, and the obtained antiserum was purified by affinity chromatography on a column with the antigen coupled to CNBr-activated SepharoseTM 4B (GE Healthcare Life Sciences, Freiburg, Germany). Bound antibodies were eluted with 0.1 M glycine, pH 2.5, and neutralized with 3 M Tris-HCl, pH 8.8 and 5 M NaCl. The specificity of purified antibodies was determined by enzyme-linked immunosorbent assay (ELISA) binding assay and immunoblotting.

### 4.4. Immunofluorescence Analysis

For immunofluorescence studies 4′,6-diamidino-2-phenylindole (DAPI) (Invitrogen, Karlsruhe, Germany) as well as F4/80 (Biolegend, Biolegend, San Diego, CA, USA), MMP9 (Abcam, Cambridge, UK), EMILIN-1 or TGFBI antibodies were used on paraformaldehyde-fixed sections and detected by corresponding secondary antibodies coupled to Cy3 (Jackson Immuno Research, Ely, UK). Images were analyzed by immunofluorescence microscopy (Nikon Eclipse TE2000-U Microscope, Tokyo, Japan).

### 4.5. Immunoblot Analysis

Skin and wound tissue samples were pulverized and lysed as described previously [14] and similar amounts were subjected to immunoblotting. Primary antibodies detecting actin (Merck-Millipore, Darmstadt, Germany), MMP9 (Abcam, Cambridge, UK), EMILIN-1, TGFBI, netrin-1 [56] and gliomedin [93] were detected with corresponding secondary antibodies coupled to horseradish peroxidase (DAKO, Agilent Technologies, Hamburg, Germany) and visualized using Amersham ECL Prime Western Blotting Detection Reagent (GE Healthcare, Freiburg, Germany).

## 5. Conclusions

**Figure 6:**
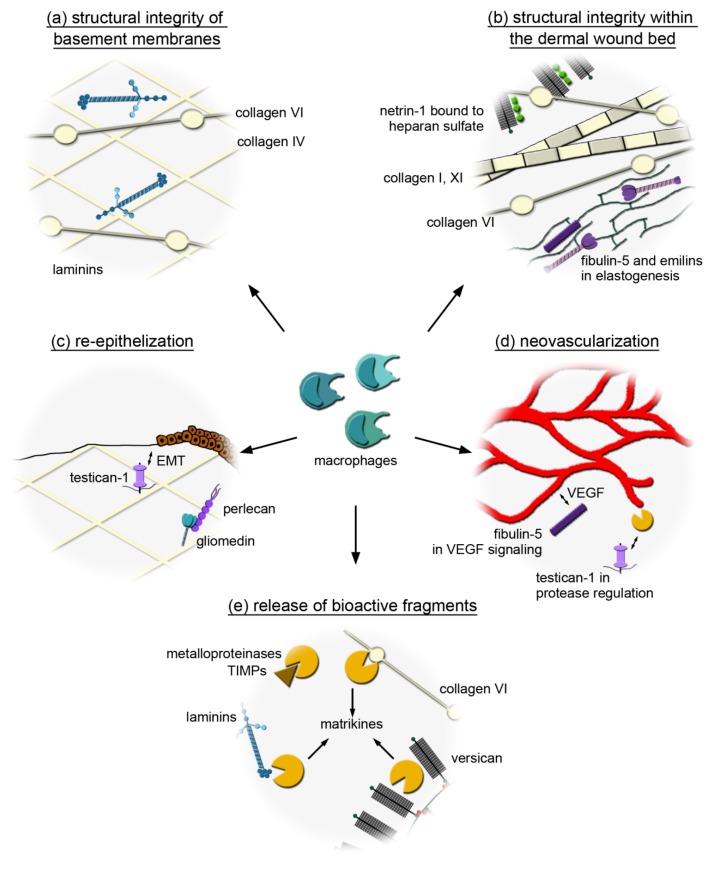
Potential wound healing mechanisms involving the macrophage-derived extracellular matrix (ECM). (**a**) Macrophages could produce collagen IV, VI and laminins to provide their own substrate and/or bridging proteins for their adhesion to basement memranes. (**b**) Macrophages may promote the formation of a microfibrillar network within the wound by producing the beaded filament forming collagen VI. Collagen XI may be utilized by macrophages to interact with fibrillar collagens and support their organization while macrophage-derived netrin-1 may provide guidance signals to organize the wound. Macrophages may secrete fibulin-5 and EMILINs to act as a scaffold protein that organize and link elastic fibres. (**c**) Macrophage-derived gliomedin that binds to perlecan might participate in basement membrane formation/stabilization and influence keratinocytes to regulate epithelial wound closure. By producing testican-1, which is a target of TGF-β and induces epithelial-to-mesenchymal transition (EMT), macrophages may support epithelial wound closure. (**d**) Macrophage-derived testican-1 may also regulate metalloproteinase activity to promote the degradation of provisional wound matrix supporting vascularization, while fibulin-5 synthetized by macrophages may regulate angiogenesis by modulating VEGF signaling. (**e**) Macrophages may express proteases (A disintegrin and metalloproteinase with thrombospondin motifs (ADAMTS2), MMP9, MMP1) and their inhibitors (TIMP1–3) to generate bioactive ECM fragments (matrikines) and may also be a source of ECM molecules that harbor these bioactive fragments (collagen VI, laminins, versican).

In summary, we define the expression profile of core matrisome and matrisome-associated genes in macrophages. While (myo) fibroblasts are considered the major source of structural ECM components in the wound, we show that macrophages also express ECM genes in vitro and in vivo that potentially contribute to the structural support of wound tissue (Figure 6a,b) as well as to re-epithelization (Figure 6c) or vascularization (Figure 6d). Other components of the macrophage secretome, such as proteases or cytokines and growth factors, are known to promote ECM remodeling or recruitment and activation of lymphocytes, fibroblasts or endothelial cells within the wound. We show that macrophages can be a source of proteases and ECM substrates that are important for the release of bioactive ECM fragments and may thereby modulate the skin wounding response (Figure 6e). Although the in vitro analysis does not accurately reflect the responses of macrophages to the more complex cellular, ECM, and signaling milieu of the in vivo wound bed, we have shown that several of the identified matrisome components are found within the wounds when macrophages represent the major cellular component. Therefore, we provide a comprehensive matrisome data set of macrophage-related genes which can be used for targeted in-depth analysis of macrophage-specific ECM networks within the wound and identification of targets for therapeutic interventions in the future.

## Supplementary Materials

Supplementary materials can be found at https://www.mdpi.com/1422-0067/20/20/5086/s1. Table S1. Expressed and regulated genes in macrophages.

## Abbreviations

ADAM	A disintegrin and metalloproteinase
ADAMTS	ADAM with thrombospondin motifs
ADAMTSL	ADAMTS like
AGRN	Agrin
A2M	Alpha-2-macroglobulin
ANXA	Annexin
BMP	Bone morphogenetic protein
BRAL	Brain link protein
CILP	Cartilage intermediate layer protein
CTS	Cathepsin
CXCL	Chemokine (C-X-C motif) ligand
CCL	Chemokine ligand
CHADL	Chondroadherin-like
CD	Cluster of differentiation
COL	Collagen
C1Q	Complement component 1q
CLEC	C-type lectin domain family
CST	Cystatin
Dexa	Dexamethasone
EMILIN	Elastin microfibril interfacer
EMT	Epithelial-to-mesenchymal transition
ECM	Extracellular matrix
FAM	Family with sequence similarity
FGL	Fibrinogen-like
FGF	Fibroblast growth factor
FBLN	Fibulin
FCN	Ficolin
FC	Fold change
FDR	False discovery rate
GLDN	Gliomedin
GREM	Gremlin
GDF	Growth/differentiation factor
HTR	High-temperature requirement
HAPLN	Hyaluronan and proteoglycan link protein
HA	Hyaluronan
HYAL	Hyaluronidase
INHB	Inhibin subunit beta
IGF	Insulin-like growth factor
ITGA	Integrin alpha subunit
ITGB	Integrin beta subunit
IFNγ	Interferon gamma
IL	Interleukin
LAMA	Laminin alpha chain
LAMB	Laminin beta chain
LAMC	Laminin gamma chain
LGALS	Lectin, galactoside-binding, soluble
LPS	Lipopolysaccharides
LH	Lysyl hydroxylase
MCSF	Macrophage colony-stimulating factor
MMP	Matrix metalloproteinase
MFGE	Milk fat globule epidermal growth fator
MDM	Monocyte-derived macrophage
NTN	Netrin
ON	Osteonectin
PLAU	Plasminogen activator, urokinase
PDGF	Platelet-derived growth factor
PLXN	Plexin
PLXDC	Plexin domain containing
PCOLCE	Procollagen C-endopeptidase enhancer
PPBP	Pro-platelet basic protein
SPP1	Secreted phosphoprotein 1
SPARC	Secreted protein acidic and rich in cysteines
SEMA	Semaphorin
SPOCK	SPARC/osteonectin, Cwcv, and Kazal-like domains proteoglycan
SRPX	Sushi repeat-containing protein
THBS	Thrombospondin
TIMP	Tissue inhibitors of metalloproteinases
TLR	Toll-like receptor
TGFβ	Transforming growth factor beta
TGFBI	Transforming growth factor beta induced
TINAGL1	Tubulointerstitial nephritis antigen-like
TSG	Tumor necrosis factor stimulated gene
TNFAIP	Tumor necrosis factor alpha induced protein
TAM	Tumor-associated macrophage
UNC	Uncoordinated locomotion
VEGF	Vascular-endothelial growth factor
VCAN	Versican
